# Bone marrow-derived mesenchymal stem cells overexpressing miR-21 efficiently repair myocardial damage in rats

**DOI:** 10.18632/oncotarget.16254

**Published:** 2017-03-16

**Authors:** Yan-Ling Zeng, Hao Zheng, Qiu-Ru Chen, Xiao-Hong Yuan, Jin-Hua Ren, Xiao-Feng Luo, Ping Chen, Zhe-Yao Lin, Shao-Zhen Chen, Xue-Qiong Wu, Min Xiao, Yong-Quan Chen, Zhi-Zhe Chen, Jian-Da Hu, Ting Yang

**Affiliations:** ^1^ Department of Hematology, Fujian Institute of Hematology, Fujian Provincial Key Laboratory of Hematology, Fujian Medical University Union Hospital, Fuzhou 350001, P. R. China; ^2^ Department of Hematology, Affiliated Nanping First Hospital of Fujian Medical University, Nanping 353000, P. R. China

**Keywords:** microRNA-21, lentiviral vector, bone marrow derived mesenchymal stem cell, anthracycline, cardiac damage

## Abstract

**Objective:**

We investigated the ability of bone marrow derived mesenchymal stem cells (BMSCs) overexpressing microRNA-21 (miR-21) to repair cardiac damage induced by anthracyclines in rats.

**Methods:**

Sprague-Dawley (SD) rats of 2~3 weeks old were selected to isolate and culture BMSCs. A lentivirus harboring pLVX-miR-21 was generated and transfected into rat BMSCs. The rats were assigned into an untreated negative control group, and groups injected with adriamycin alone or with adriamycin followed by BMSCs, pLVX-BMSCs or pLVX-miR-21-BMSCs (n = 10 each). Proliferation and migration of cells were detected by cholecystokinin-8 (CCK- 8) and transwell. MiR-21 expression, mRNA expressions of B cell lymphoma 2 (Bcl2), BAX (BCL-2-associated X protein) and vascular endothelial growth factor (VEGF) were tested by qRT-PCR. Western blotting was applied to detect protein expressions of Bcl-2, Bax and VEGF.

**Results:**

Using CCK- 8 and transwell assays, we found that pLVX-miR-21-BMSCs, which overexpressed miR-21, exhibited greater proliferation and migration than untransfected BMSCs or pLVX-BMSCs. Ultrasonic cardiograms and immunohistochemical analysis demonstrated that among the five groups, the pLVX-miR-21-BMSC group exhibited the most improved heart function and enhanced angiogenesis. Moreover, the pLVX-miR-21-BMSC group showed enhanced expression of Bcl-2, VEGF and Cx43 and reduced expression of Bax, BNP and troponin T.

**Conclusion:**

These findings suggest miR-21 overexpression enhanced the proliferation, invasiveness and differentiation of BMSCs as well as expression of key factors (Bcl-2, VEGF and Bax) essential for repairing the cardiac damage induced by anthracyclines and restoring heart function.

## INTRODUCTION

Anthracyclines are effective drugs against various hematologic diseases and malignancies [1]. However, their clinical use is restrained by the cumulative dose-dependent cardiotoxicity that can result in irreversible chronic cardiac injury and congestive heart failure [2, 3]. Mesenchymal stem cells (MSCs) are pluripotent stem cells with strong self-renewal and differentiation ability that are used in transplantation cell therapy [4, 5]. MSCs can differentiate into myocardial cells and produce vascular endothelial growth factor (VEGF) and anti-apoptotic factors that can improve myocardial function [6, 7]. The bone marrow-derived mesenchymal stem cells (BMSCs) are the most common MSCs that are used therapeutically in various diseases including myocardial injury because they are easily extractable, survive better after transplantation and show weak immunogenicity [8]. The BMSCs differentiate into cardiomyocytes and replace the apoptotic myocardium thereby improving the function of damaged hearts [9]. They are critical for immunomodulatory function, inflammatory response and epithelial function [10]. However, the poor long-term survival of transplanted MSCs presents a major therapeutic problem that needs to be overcome [11]. Therefore, various factors that can enhance long term survival of MSCs need to be identified and one such category includes the microRNAs. In fact, several upregulated miRNAs were shown to regulate differentiation of BMSCs [12].

MicroRNAs (miRNAs) are non-coding RNAs that are 18 to 25 nucleotides long, which are involved in cardiac development and the process of cardiac hypertrophy [13]. MiR-21 is an oncogenic miRNA that is over-expressed in many solid tumors and shown to down-regulate tumor suppressors, such as phosphatase and tensin homolog (PTEN), B-cell lymphoma-2 (bcl-2) and tropomyosin l (TPM1) [14–16]. In addition, miR-21 was associated with cellular proliferation, migration and apoptosis [17]. It was highly expressed in cardiomyocytes and played a crucial role in myocardial development and disease [18]. MiR-21 demonstrated anti-apoptotic function during cardiomyocyte apoptosis induced by ischemia and hypoxia [19]. Nevertheless, the role of miR-21 in anthracycline-induced cardiac injury is unknown. Therefore, in this study, we investigated the role of miR-21-overexpressing BMSCs in repairing anthracycline-induced cardiac damage.

## RESULTS

### Morphological and phenotypic characteristics of the rat BMSCs

**Figure 1:**
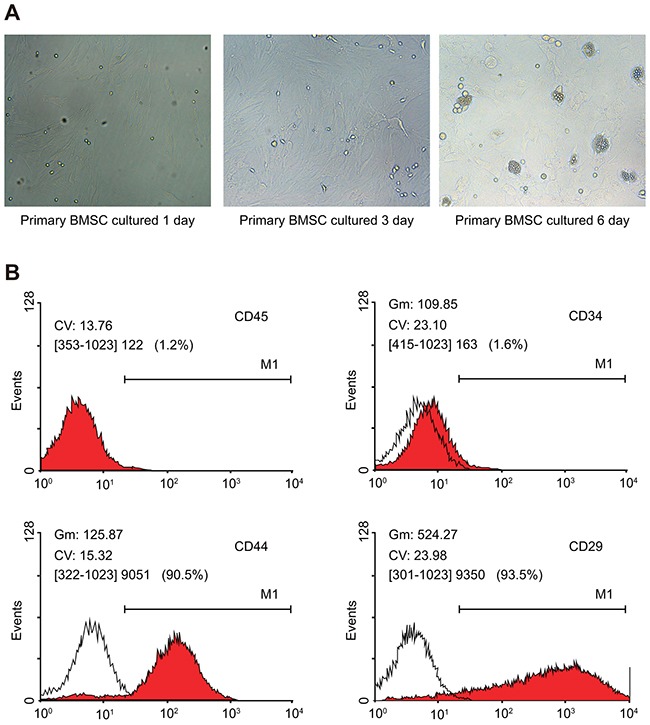
Characterization of morphological changes and phenotype of rat BMSCs **(A)** Morphological changes of BMSCs (× 200); **(B)** FACS analysis of surface markers on BMSCs.

Initially, 95% of the rat BMSCs attached in 24h after seeding with only fewer cells attaining polygonal and fusiform shapes. By the 3^rd^ day, the BMSCs formed small colonies and exhibited logarithmic growth. The cell attachment rate was nearly 90% by the 6^th^ day and the cells exhibited polygonal arrangement. Further, the cells showed no significant differences in cellular morphology upon passage (Figure 1A). Flow cytometry showed that 1.3%, 1.7%, 90.7% and 93.3% BMSCs positively expressed CD45, CD34, CD44 and CD29, respectively (Figure 1B).

### Lentivirus infection and over-expression of miR-21 in rat BMSCs

**Figure 2:**
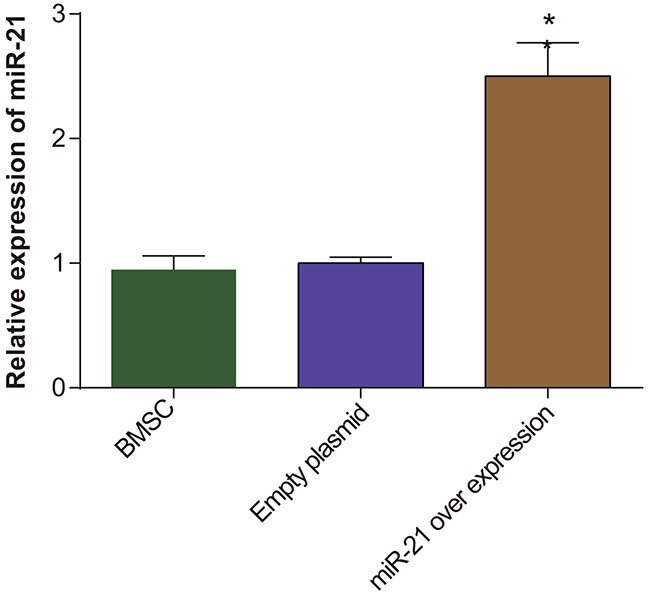
The miR-21 expression in BMSC, pLVX-BMSC and pLVX-miR-21-BMSC groups Note: * denotes *P* < 0.05.

We tested the expression of miR-21 in the transfected BMSCs by qRT-PCR. We observed that miR-21 expression was highest in the pLVX-miR-21-BMSCs compared to BMSCs alone and pLVX-BMSCs (*P* < 0.05; Figure 2). This analysis demonstrated that pLVX-miR-21 had successfully infected the BMSCs and was ready for further experiments (Figure 2).

### MiR-21 overexpression enhances migration of rat BMSCs as determined by Transwell assay

**Figure 3:**
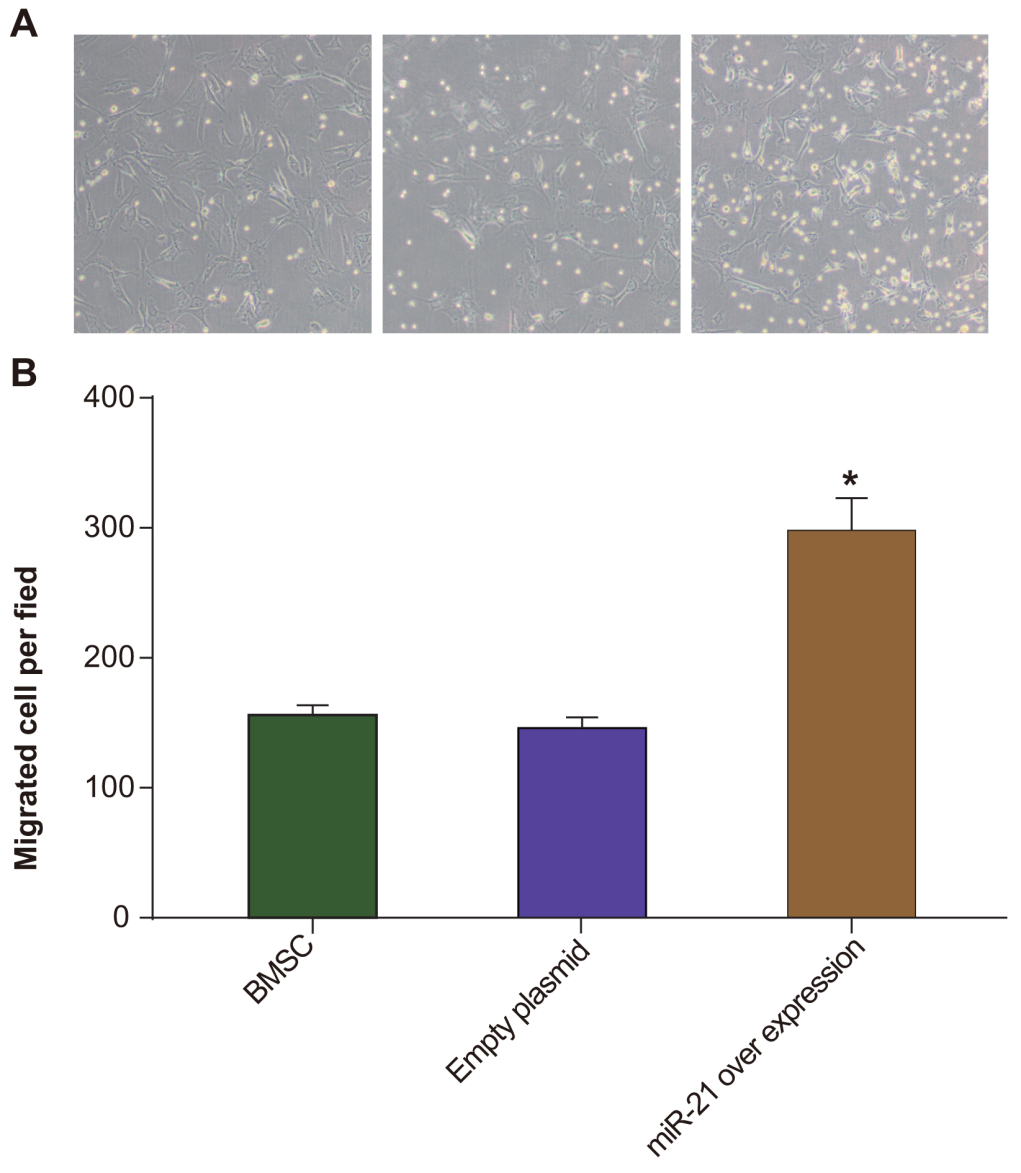
Transwell migration assay of rat BMSCs **(A)** Transwell assay was used to compare the migration of BMSC, pLVX-BMSC and pLVX-miR-21-BMSC groups. The blue spots point to the BMSCs that migrated to the lower chamber in transwell; ruler 200μm; **(B)** Total number of cells counted per field in BMSC, pLVX-BMSC and pLVX-miR-21-BMSC groups. * denotes *P* < 0.05 comparing BMSC, pLVX-BMSC and pLVX-miR-21-BMSC groups.

To assess the effects of miR21 on migration of BMSCs, we conducted *in vitro* transwell assay and found that pLVX-miR-21-BMSCs migrated nearly 2 fold greater (298 ± 25.0 cells per field) than the control BMSCs and pLVX-BMSCs (146 ± 8.10 cells per field in both groups; *P* > 0.05; Figure 3). This indicated that miR-21 overexpression enhanced the migration of BMSCs.

### MiR-21 overexpression promotes BMSC proliferation of BMSCs

**Figure 4:**
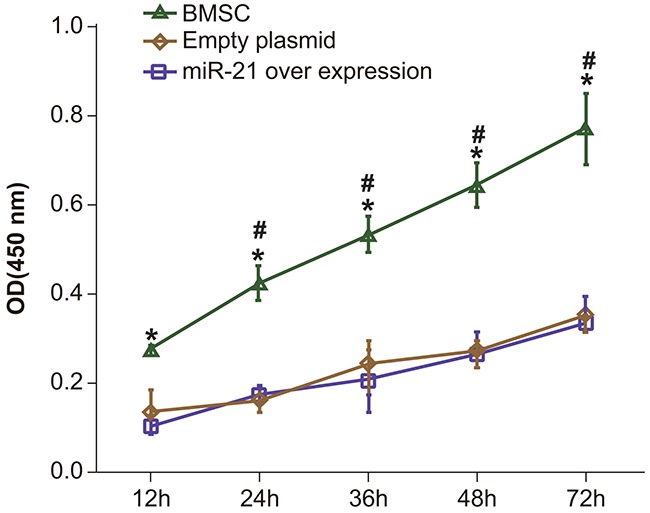
Comparing BMSC proliferation by CCK-8 assay between BMSC, pLVX-BMSC and pLVX-miR-21-BMSC groups Note: * denotes *P* < 0.05; # denotes *P* < 0.05 when compared with the former time point.

Next, we assessed the effect of miR-21 on BMSC proliferation by the CCK-8 assay. Our data demonstrated that there were no significant differences in proliferation of the control BMSCs and the pLVX-BMSCs (*P* > 0.05). However, the pLVX-miR-21-BMSCs exhibited increased proliferation at all time points (12, 24, 36, 48 and 72h) compared to control BMSCs and the pLVX-BMSCs (Figure 4; *P* < 0.05). Further, among all the three groups, cell proliferation increased in relation to time as expected (Figure 4). Therefore, the CCK-8 assay results demonstrated that miR-21 over-expression promoted proliferation of BMSCs.

### Mir-21 overexpressing BMSCs improve heart function in myocardial injury model rats

We then evaluated the myocardium injury model by assessing heart function by ultrasonic cardiogram (UCG) analysis. We observed that the EF, FS, LVEDD and LVESD values for heart function in the negative control group were higher than the other four groups (adriamycin treatment only, BMSC, pLVX-BMSC and pLVX-miR-21-BMSC) before transfection (*P* < 0.05; Table 1). This showed that adriamycin treatment had successfully induced myocardium injury. When heart function was evaluated after 28 days, the EF, FS, LVEDD and LVESD values were highest in the negative control group followed by pLVX-miR-21-BMSC group whereas it was lower in the BMSC and pLVX-BMSC groups and least in the adriamycin treatment only group (*P* < 0.05; Table 1). Also, the EF, FS, LVEDD and LVESD values did not change significantly for the control and the adriamycin groups before and after transfection (*P* > 0.05). However, the EF, FS, LVEDD and LVESD values were greater in the BMSC, pLVX-BMSC and pLVX-miR-21-BMSC groups after transfection compared to those before transfection suggesting that the BMSCs helped recovery from the myocardial injury (*P* < 0.05; Table 1).

**Table 1:** Comparisons on values of heart function before and after animal model establishment

		Control group	Adriamycin group	BMSCs group	NC group	miR-21 overexpression group
Before transfection	EF (%)	84.59 ± 1.5	41.09 ± 2.3*	41.29 ± 2.7*	41.12 ± 2.78*	42.09 ± 2.9*
	FS (%)	47.9 ± 2.4	15.78 ± 1.32*	16.69 ± 1.3*	16.78 ± 1.35*	17.5 ± 1.2*
	LVEDD (mm)	9.86 ± 0.05	4.11 ± 0.16*	4.02 ± 0.08*	3.99 ± 0.08*	4.17 ± 0.2*
	LVESD (mm)	10.37 ± 0.15	4.01 ± 0.09*	3.99 ± 0.11*	4.00 ± 0.08*	4.08 ± 0.10*
After transfection	EF (%)	83.79 ± 1.66	40.97 ± 2.18*	49.8 ± 3.5^*#△^	49.0 ± 3.3^*#△^	75.9 ± 1.6^*#&△^
	FS (%)	47.8 ± 2.41	16.21 ± 1.24*	24. 4 ± 1.2^*#△^	24.9 ± 1.3^*#△^	38.8 ± 2^*#&△^
	LVEDD (mm)	9.91 ± 0.05	4.15 ± 0.16*	6.12 ± 0.13^*#△^	6.13 ± 0.19^*#△^	8.0 ± 0.1^*#&△^
	LVESD (mm)	10.40 ± 0.14	4.14 ± 0.11*	6.12 ± 0.7^*#△^	6.10 ± 0.1^*#△^	8.01 ± 0.5^*#&△^

Note: EF, ejection fraction; FS, Fraction shortening; LVEDD, Left ventricular end-diastolic dimension; LVESD, left ventricular end systolic diameter *, *P* < 0.05, compared with control group;^#^, *P* < 0.05, compared with Adriamycin group; ^&^, *P* < 0.05, compared with BMSCs group and NC group; ^△^, *P* < 0.05, compared with before transfection; NC, negative control; BMSCs, bone marrow mesenchymal stem cells; miR-21, microRNA-21.

### MiR-21 overexpressing BMSCs are most efficient in restoring myocardial function following adriamycin injury

**Figure 5:**
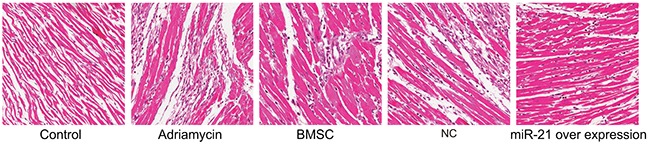
H&E stained myocardial tissue of rats post recovery among the five groups (Negative control, adriamycin treatment only, BMSC, pLVX-BMSC and pLVX-miR-21-BMSC; x 200)

To further analyze if the miR-21 overexpressing BMSCs were efficient in repairing myocardial injury, we analyzed the H&E stained myocardial tissue sections from the different treatment groups. We observed that the myocardial tissue in the negative control group was normal and well compacted without any scar tissue of myocardial infarction (Figure 5). On the other hand, in the adriamycin treatment only group, cardiomyocytes disappeared leaving behind large areas of scar tissue indicating myocardial infarction. Further, we observed large areas of myocardial tissue that was necrotic and fibrotic interspersed with small islands of normal myocardial tissue (Figure 5). The scar areas in the BMSC, pLVX-BMSC and pLVX-miR-21-BMSC groups were significantly smaller compared to the adriamycin group showing that BMSCs helped recovery of myocardial tissue from injury (Figure 5). Further, the pLVX-miR-21-BMSC group demonstrated maximal recovery among the three groups (Figure 5). These data therefore indicated that adriamycin induced significant myocardial tissue injury that was repaired by the BMSCs, of which the pLVX-miR-21-BMSC group showed the most significant recovery showing the beneficial effects of miR-21.

### Mir-21 overexpressing BMSCs induce significant angiogenesis during myocardial recovery from injury

**Figure 6:**
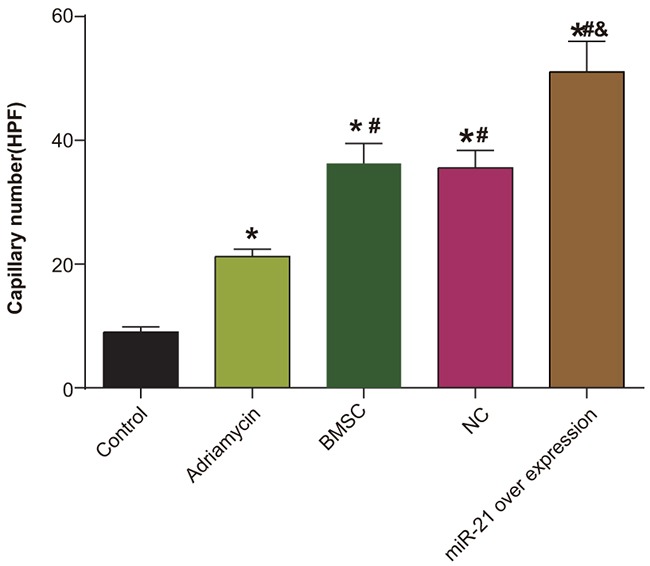
Immunohostochemical analysis of Factor VIII demonstrating angiogenesis density in the five groups of rats (negative control, adriamycin treatment only, BMSC, pLVX-BMSC and pLVX-miR-21-BMSC groups) * denotes *P* < 0.05, compared with the negative control group; ^#^ denotes *P* < 0.05, compared with the adriamycin treatment only group; ^&^ denotes *P* < 0.05, compared with the BMSCs group.

Further, we evaluated angiogenesis in the myocardial injury model by immunohistochemical analysis of Factor VIII. We observed that Factor VIII appeared as brown granules in the cell nucleus, mainly distributed in the vascular endothelium (Figure 6). Among the different treatment groups, the negative control group had no obvious angiogenesis, whereas the adriamycin treatment only group demonstrated low amount of angiogenesis. In the BMSC, pLVX-BMSC and pLVX-miR-21-BMSC groups, significantly higher angiogenesis was observed in the damaged area of myocardial tissue (Figure 6). In the pLVX-miR-21-BMSC group that demonstrated most angiogenesis, regular patterned arrangement between the survived myocardial cells and the transplanted cells was observed (Figure 6). Also, enhanced angiogenesis was observed in the margins.

### Mir-21 overexpressing BMSC group demonstrates enhanced Bcl-2 and VEGF and decreased Bax levels

**Figure 7:**
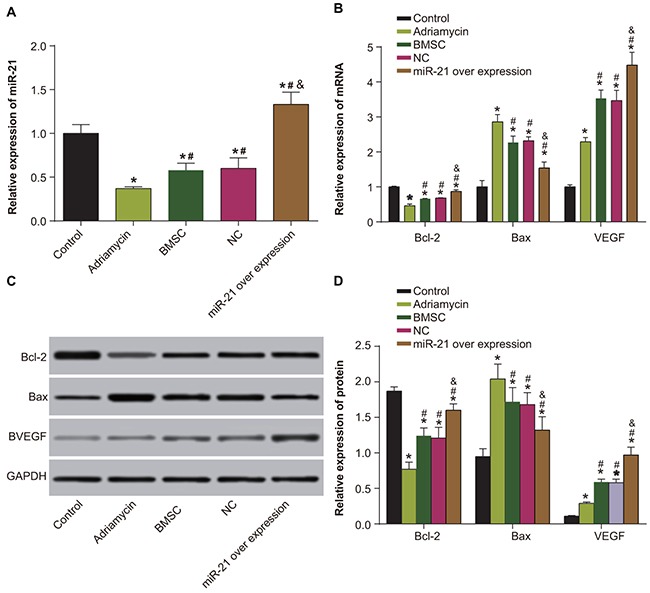
Expression of miR-21, VEGF, Bcl-2 and Bax among five groups of rats (negative control, adriamycin treatment only, BMSC, pLVX-BMSC and pLVX-miR-21-BMSC) **(A)** MiR-21 expression detected by qRT-PCR; **(B)** Expression of Bcl-2, Bax and VEGF detected by qRT-PCR; **(C)** Expression of Bcl-2, Bax and VEGF detected by Western blotting; **(D)** Relative expression of Bcl-2, Bax and VEGF detected by Western blotting; Note: * denotes *P* < 0.05, compared with the negative control group; ^#^ denotes *P* < 0.05, compared with the adriamycin treatment group; ^&^ denotes *P* < 0.05, compared with the BMSCs and the pLVX-BMSC group; qRT-PCR, quantitative real-time polymerase chain reaction.

To understand the molecular details regarding repair from myocardial injury, we analyzed the the expression of miR21, VEGF, Bcl-2 and Bax by qRT-PCR. Our data showed that whereas miR-21 expression significantly decreased in the adriamycin treatment only, the BMSCs and the pLVX-BMSC groups, it was significantly higher in the pLVX-miR-21-BMSC group (*P* < 0.05). The miR-21 expression was least in the adriamycin group and highest in the pLVX-miR-21-BMSC group (*P* < 0.05). Further, compared with the control group, the remaining four groups demonstrated decreased Bcl-2 expression and increased expression of Bax and VEGF (all *P* < 0.05). The pLVX-miR-21-BMSC group demonstrated highest Bcl-2 and VEGF expression and least Bax expression compared to the other groups (*P* < 0.05; Figure 7). These results showed that miR-21 overexpression promoted most efficient expression of relevant factors that was necessary for recovery from myocardial damage.

### MiR-21 overexpressing BMSCs show elevated Cx43 and reduced troponin T and BNP levels

**Figure 8:**
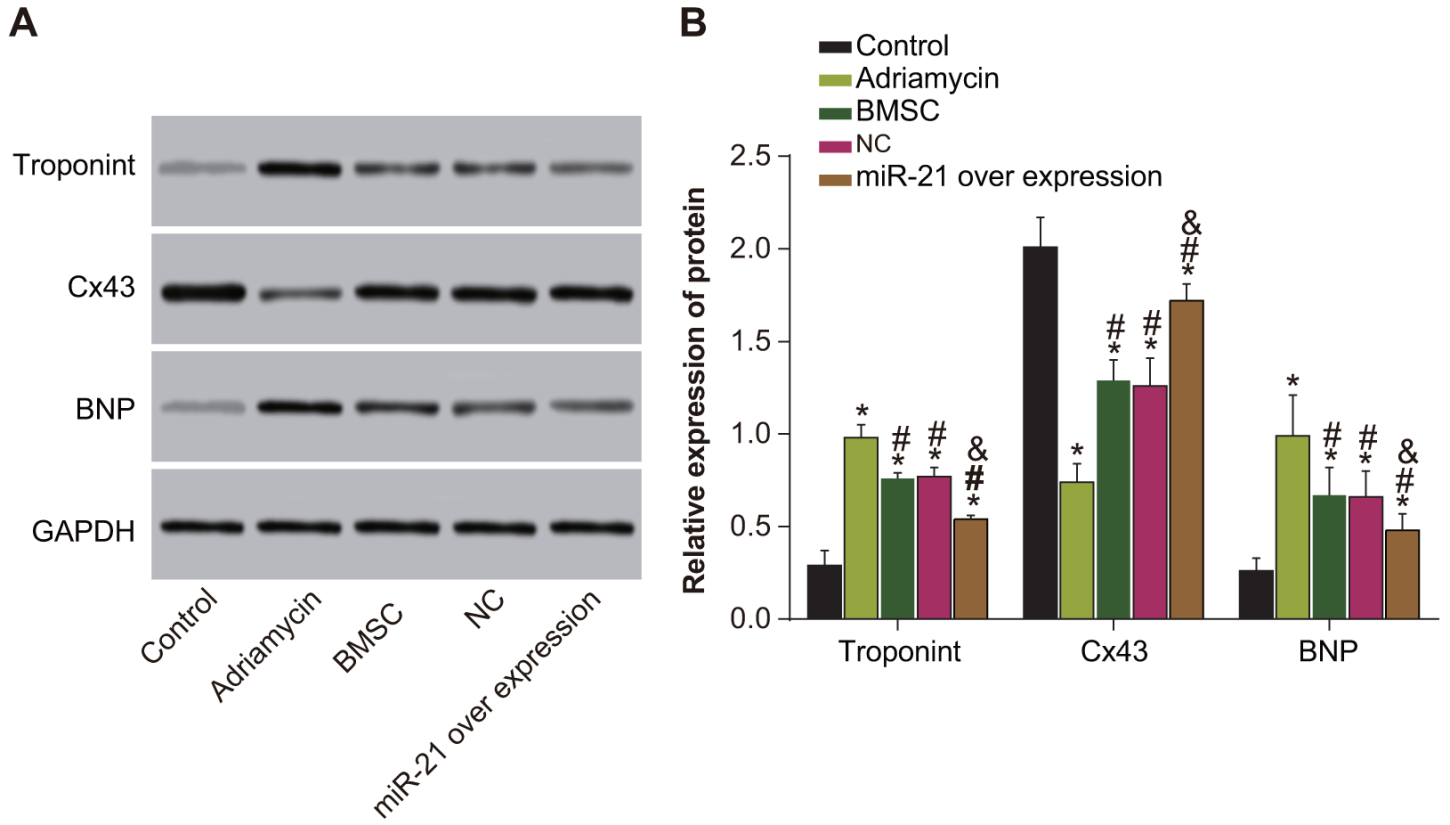
Expressions of Cx43, troponin T and BNP among five groups of rats (negative control, adriamycin treatment only, BMSC, pLVX-BMSC and pLVX-miR-21-BMSC) **(A)** Expression of Cx43, troponin T and BNP detected by Western blotting; **(B)** Relative expression of Cx43, troponin T and BNP and comparison between groups. Note: * denotes *P* < 0.05, compared with the control group; ^#^, *P* < 0.05, compared with the adriamycin treatment only group; ^&^ denotes *P* < 0.05, compared with the BMSCs and the pLVX-BMSC group.

Western blotting data showed decreased Cx43 and elevated troponin T and BNP levels in the adriamycin treatment only, the BMSCs, pLVX-BMSC and pLVX-miR-21-BMSC groups compared to the negative control group (*P* < 0.05; Figure 8). However, in comparison to the adriamycin treatment only group, we observed elevated Cx43 expressions and reduced troponin T and BNP levels in the BMSCs, pLVX-BMSC and pLVX-miR-21-BMSC groups (*P* < 0.05; Figure 8). Further, the pLVX-miR-21-BMSC group had highest Cx43 and least troponin T and BNP among the groups levels among adriamycin treatment only, the BMSCs, pLVX-BMSC and pLVX-miR-21-BMSC groups (*P* < 0.05; Figure 8). These results showed that myocardial tissue in miR-21-modified BMSC group with high Cx43 and low troponin T and BNP levels recovered most efficiently than the other groups.

## DISCUSSION

Recently, miRNA have been identified as critical molecular targets in the process of myocardial injury and recovery [20–22]. Our study demonstrates that miR-21 overexpression contributes to enhanced invasion and proliferation of BMSCs, thereby significantly enhancing their ability to repair myocardial injury by anthracyclines.

Previous studies showed that miR-21 had a key role in the development and progression of human cancers [23–25]. MiR-21 induced cell proliferation and invasion in esophageal squamous cell carcinoma and colorectal cancer [26, 27]. Further, previous studies demonstrated that miRNAs were associated with BMSC proliferation and cardiomyocyte differentiation, consistent with our data [28–31]. Trohatou and colleagues showed that miR-21 modulated Sox2 expression in the MSCs contributed to their proliferation and differentiation [32].

Our data also demonstrated that the miR-21 over-expressing BMSCs enhanced cardiac function, reduced scar area and promoted significant angiogenesis. Recently, miR-21 was implicated in protecting the ischemic myocardium from apoptosis, which was important in the early stages of acute myocardial infarction [33]. Further, since miR21 overexpressing BMSCs effectively eliminated scars, which involve change of appearance and histopathology of normal skin, miR-21 may be beneficial in future clinical treatment of skin scars [34].

Regulation of angiogenesis includes proliferation and migration of endothelial cells and involves growth factors such as VEGF [35]. Previously, miR-21 was instrumental in angiogenic mediation of ischemic milieu and orchestration of the therapeutic potential of human multipotent cardiovascular progenitors [36]. Our study similarly demonstrated that miR-21-overexpressing BMSCs showed enhanced expression of Bcl-2, VEGF and Cx43 and reduced expression of Bax, BNP and troponinT. BMSCs had been shown to improve heart function by their ability to proliferate and induce angiogenesis resulting in myogenic cell differentiation [37]. Therefore, transplanted MSCs potentially could differentiate into vasculature cells and cardiomyocytes and recruit endogenous cardiac stem cells apart from secreting many different paracrine factors [38, 39]. García and co-workers confirmed that miR-21 participated in pressure overload-associated myocardial remodeling [40]. Further, Wang and colleagues showed that exosomal miR-21 enhanced the cardio-protection properties of human MSCs [41]. Apoptosis is critical for development and maintenance of tissues and pro-apoptotic protein Bax and the anti-apoptotic protein Bcl-2 function as important regulators during these processes [42]. Further, the Cx43 channels are essential for electrical coupling and direct cardiac cell to cell communication that ensures heart function and are involved in adaptation of the heart to irradiation-induced injury in co-ordination with miR-21 [43]. Also, BNP and troponinT are biomarkers for cardiac injury [44, 45]. Therefore, our study showed that the miR21 overexpressing BMSCs efficiently regulated key factors that were essential in repairing the myocardial injury. However, the intrinsic role of miR-21 on cardiac damage is unclear and further studies are necessary to better understand its functional role during cardiac damage in response to anthracycline

In conclusion, our study demonstrates that miR-21 overexpression enhances the proliferation, invasiveness and differentiation of the BMSCs apart from efficiently regulating key factors that are essential in inhibiting apoptosis of cardiomyocytes and repairing the cardiac damage induced by anthracyclines.

## MATERIALS AND METHODS

### Ethics statement

Animal experiments were conducted in strict accordance with the approved animal protocols and guidelines established by Medicine Ethics Review Committee for animal experiments of Fujian Institute of Hematology, Fujian Provincial Key Laboratory of Hematology, Fujian Medical University Union Hospital.

### Isolation and characterization of BMSCs

To obtain rat BMSCs, 2-3 week old Sprague-Dawley (SD) rats from the Laboratory of Molecular Biology of Shandong University (Shandong province, China.) were sacrificed by cervical dislocation and both their both hind limbs were isolated. Further, the bone marrow cavity of the hind limbs was flushed with DMEM medium (Solarbio, Shanghai, China) to isolate total bone marrow cells. The cells (primary P_0_ BMSCs) were then centrifuged and aseptically cultured in DMEM medium containing 2x antibiotics (Pen-Strep) and 10% fetal calf at 37°C with 5% CO_2_. Fresh culture medium was added every 24-48 h. Once the cells reached about 90% confluence (in 10 days), the P_0_ cells were digested with 0.25% trypsin (Solarbio, Shanghai, China), terminated with FBS and passaged at a ratio of 1: 2. For phenotypic characterization, the P_4_ BMSCs were trypsinized, centrifuged and then incubated with anti- CD34, -CD45, -CD29 and -CD44 antibodies in PBS with 1% fetal calf serum for 30 min at 4°C followed by flow cytometry.

### Construction and identification of lentiviral vector

To clone miR-21, we first designed primers based on the rat miR-21 sequence (rno-miR-21-5p, MIMAT0000790, UAGUUAUCAGUGAUGUUA). The primers were: forward: 5′-CGGGATCCAGCCACTACCAAGGCATGTT-3′; reverse: 5′-CGGAATTCAACCACGACTAGAGGCTGAC-3′ (Invitrogen, Shanghai, China). Then, miR-21 with flanking sequences was PCR amplified as follows: The amplified miR-21 PCR fragment was then recombined into empty vector pLVX-shRNA2 (Laboratory of Molecular Biology, Shandong University, China). Further, the DH5α competent cells (Solarbio, Shanghai, China) were transformed with the recombinant vector containing miR-21 followed by selection of recombinant clones. The positive clones were then verified by extracting the plasmid DNA from positive clones followed by PCR amplification of miR21 and sequencing (Invitrogen, Shanghai, China).

To generate the lentivirus, recombinant plasmid DNA containing miR21 was packaged into 293T cells (Laboratory of Molecular Biology of Shandong University, Shandon, China) using the calcium phosphate method. After 48h, the supernatant containing the virus was collected, filtered by millipore filter (0.45 μm) and concentrated by ultracentrifugation. Finally, the miR-21 (pLVX- miR-21) lentivirus was re-suspended in cold phosphate buffer saline (PBS).

### BMSCs transfection

The P_4_ BMSCs were grown in Dulbecco's modified Eagles Medium (DMEM) medium with 10% FBS in a 6-well plate until they reached 70% confluence. At this point, the BMSCs were divided into three groups (in triplicate), namely, BMSC only (no transfection), BMSC plus empty pLVX (transfected with empty plasmid) and BMSC plus pLVX-miR-21 (transfected with miR-21 over-expression plasmid). Then, pLVX (empty) or pLVX-miR-21virus in combination with 8μg/ml polybrene (Solarbio, Shanghai, China) was transfected into corresponding BMSCs. For the negative control group, BMSCs were transfected with 8μg/ml polybrene alone. Further, the conditioned DMEM medium of transfected BMSCs was collected after 24h and preserved at −80°C.

### CCK-8 assay

The transfected cells (100 μl/well) from the three experimental groups described above were seeded in a 96-well plate at 37°C. 10μl of CCK-8 reagent (Beyotime Biotechnology, Shanghai, China) was added to three wells in each group after 12, 24, 36, 48 and 72h, respectively. Then the plate was placed in the refrigerator for 2h followed by measurement of the optical density (OD) at 450nm in a micro-plate reader (Bio-Rad, California, USA). The data were plotted to obtain the growth curve. The experiment was repeated thrice.

### Transwell assay

To determine the migration rate, the BMSCs from the three groups were trypsinized, centrifuged and washed twice with PBS. Then, the cells were re-suspended in DMEM medium with 10% fetal bovine serum (FBS). Then, 100μl cell suspension with 100μl DMEM medium containing 10% FBS was added into each of the 24-well plate. The transwell chamber was then placed and the cells in the transwell chamber were incubated at 37°C for 24h. After 24h, the transwell chamber was removed and the cells were washed with PBS and fixed in 4% formic acid (Solarbio, Shanghai, China) for 10 mins followed by staining with Hoechst 33258 (lμg/ml, Solarbio, Shanghai, China) for 30 mins in dark. Finally, the cells were washed thrice with PBS and the total numbers of migrated cells were counted by microscopy.

### Animal model establishment and grouping

A total of 50 SPF grade 4 week old male SD rats (Laboratory of Molecular Biology of Shandong University, Shandong, China) that weighed 100-110g were divided into five groups: negative control group, the adriamycin group, the BMSC group, pLVX-BMSC group and the pLVX-miR-21-BMSC group. The rats in the adriamycin, BMSC, pLVX-BMSC and pLVX-miR-21-BMSC groups were intraperitoneally injected seven times with 2mg/kg adriamycin once in two days whereas the control group rats were injected with the same amount of normal saline. The BMSC, pLVX-BMSC and pLVX-miR-21-BMSC group rats were administrated with BMSCs, pLVX-BMSC and pLVX-miR-21-BMSC by intraperitoneal injection, respectively, whereas the control and adriamycin group rats were injected with same amount of normal saline and continually fed for 4 weeks.

### Ultrasonic cardiogram (UCG) detection

Heart function of each rat was evaluated by UCG (Vivid7Dimension (General Electric Company, USA) before and 28 days after transfection. Briefly, the rats were anaesthetized with isoflurane (1.7% end-tidal concentration) in 40% oxygen (GH-reagent, Shanghai, China) and the ultrasound probe was inserted into the short axis of the left thorax to collect the two-dimensional echocardiography at the papillary muscle. Based on the myocardial contractility parameters, left ventricular end diastolic diameter (LVEDD) and (left ventricular end systolic diameter (LVESD) were determined. The ejection fractions (EF) and fractional shortening (FS) were calculated using the Teichholtz formula and the mass analysis function of Argus software was used to measure and count the mean value during three cardiac cycles.

### HE staining

Once the UCG was completed 28 days post-transfection, the rats were sacrificed and the hearts were harvested from the chest cavity. The hearts were washed with PBS after flushing out all blood by squeezing. The anterior wall of the left ventricle was isolated to prepare tissue slices and preserved at −80°C. The myocardial tissues were fixed with 4% paraformaldehyde (Solarbio, Shanghai, China) for 5 min and then dried. Then, the fixed tissue was stained with hematoxylin (Solarbio, Shanghai, China) for 5 min, rinsed with pathological return blue fluid (Solarbio, Shanghai, China) for 2 min, washed with PBS and dried. Finally, tissues were rinsed in 95% ethanol for 1 min and dried before staining with eosin for 3 min. The specimens were observed under a light microscope and the pathological changes were documented.

### Immunohistochemistry

Myocardial tissue slices from rats were fixed with 0.4% paraformaldehyde (Solarbio, Shanghai, China) for 5 min. Then the slices were washed, dried and incubated with endogenous peroxidase blocking solution (Solarbio, Shanghai, China) for 10 min. After the tissues were washed with PBS, they were incubated with non-immune serum (Solarbio, Shanghai, China) for 10 min, followed by factor VIII polyclonal antibody (Solarbio, Shangai, China) for 1h and rinsed with PBS. Further, the specimens were incubated with HRP-conjugated secondary antibody (1:5000; MB005, Solarbio, Shanghai, China) at room temperature for 30 min and then washed with PBS thrice for 5 min. The specimens were treated with 3,3′-diaminobenzidine (DAB) (Solarbio, Shanghai, China) for color development and were analyzed under a light microscope. Haematoxylin was used for re-staining. The images were also analyzed for vessel density and the numbers of capillaries per field.

### Quantitative real-time polymerase chain reaction (qRT-PCR)

Total RNA of heart tissue was extracted by Trizol method using the RNA isolation kit (Solarbio, Shanghai, China) and the purity and density of RNA determined from the OD260/OD280 ratio using a UV-spectrophotometer. RNA samples were preserved at −80°C. For qRT-PCR, primers for miR-21, B cell lymphoma 2 (Bcl-2), BCL-2-associated X protein (Bax) and glyceraldehyde-3-phosphate dehydrogenase (GAPDH) were designed using the Primer5.0 software using the RNA sequences from the Genbank (Table 2) and synthesized by Shanghai Gene Pharma Co., Ltd. The reverse transcription of total RNA (30 μg) was conducted according to Reverse Transcription System A3500 kit (Promega, USA) followed by PCR on the ABI 7500 (Applied Biosystem, CA, USA). The PCR conditions were: 95°C for 3 min followed by 40 cycles of 95°C for 30s, 55°C for 30s, 72°C for 1 min, and finally, 72°C for 5 min. For quantitation, PCR recipe included pre-mix Ex-Taq or SYBR Green Mix 12.5μl, Forward Primer 1μl, Reverse Primer 1μl, DNA template 4μl and ddH_2_O up to 25μl. GAPDH was used as internal reference. The melting curve was used to evaluate the credibility of PCR results. Ct value (point at which the amplification curve reaches its maximal) and 2^−ΔΔCt^ were calculated to determine the relative expression of target genes [46].

**Table 2:** Primers designed for miR-21, Bcl-2, Bax, VEGF and GAPDH

PCR primerssequence	Forward (5′-3′)	Reverse (5′-3′)
miR-21	CGGGATCCAGCCACTACCAAGGCATGTT	CGGAATTCAACCACGACTAGAGGCTGAC
Bcl-2	GAAACAGATGTCCCTACCAACCAGA	TCAGCATGGCTCAAAGTGCAG
Bax	CCAGTTGAAGTTGCCGTCAGAA	GCGAGTGTCTCAAGCGCATC
VEGF	TGTGCGGGCTGCTGCAATGAT	TGTGCTGGCTTTGGTGAGGTTTGA
GAPDH	AGGGGCCATCCACAGTCTTC	AGAAGGCTGGGGCTCATTTG

Note: Bcl-2, B-cell lymphoma-2 B; Bax, Bcl-2 Associated X Protein B; VEGF, vascular endothelial growth factor; GAPDH, glyceraldehyde-phosphate dehydrogenase; miR-21, microRNA-21.

### Western blotting

Total cellular protein was extracted 48 h after transfection and the protein concentration was measured using the BCA kit (Solarbio, Shanghai, China). 30μg of total protein was boiled with 2X loading buffer at 95°C for 10 min. After that, samples were electrophoresed in 10% SDS-PAGE (Solarbio, Shanghai, China) at 80V to 120V followed by transfer onto PVDF membrane (Millipore, USA) at 100V for 45-70 min. Then, the membrane was blocked with 5% bovine serum albumin (BSA) for 1h at room temperature followed by incubation with the appropriate primary antibodies at 4°C overnight. The antibodies used were: rabbit anti human Bax antibody (A-6223-100; 1: 1000); rabbit anti human Bcl-2 antibody (MAB7733; 1: 1000); rabbit anti human VEGF antibody (MAB8733; 1: 1000); troponin T antibody (MAB1733; 1: 100); Connexin43 (Cx43) (MAB3433; 1: 100), B-type natriuretic peptide B (BNP) (MAB3564; 1: 100) and rabbit anti human GAPDH antibody (AG019; 1: 1000) that were obtained from Solarbio, Shangai, China. The blots were then rinsed thrice with TBST for 5 mins followed by incubation with HRP-conjugated goat anti-rabbit secondary antibody (MB005, Solarbio, Shanghai, China; 1:5000) at room temperature for 1h. After washing the membrane thrice for 5 mins each with TBST, the blots were developed by chemiluminescence followed by imaging with the Bio-rad geldoc EZ formatter (Bio-rad, California, USA). The ratio of the gray value between the target and the reference band was used to calculate the relative expression of proteins using the Image J software (Bio-rad, California, USA).

### Statistical analysis

All the data were statistically analyzed by the SPSS 21.0 software. The data are expressed as mean ± standard deviation. Statistical significance of the differences between the various experimental groups were determined by either the paired t-test or one-way ANOVA or LSD-t-test (LSD = least significant difference). *P* < 0.05 was considered statistically significant.
